# Influence of sperm DNA fragmentation on the clinical outcome of *in vitro* fertilization-embryo transfer (IVF-ET)

**DOI:** 10.3389/fendo.2022.945242

**Published:** 2022-07-14

**Authors:** Chunhui Zhu, Fang Chen, Shengmin Zhang, Hong She, Yun Ju, Xidong Wen, Chunxia Yang, Yan Sun, Naijun Dong, Tongmin Xue, Kaifeng Liu, Feng Li, Hengmi Cui

**Affiliations:** ^1^ Department of Reproductive Medicine Center, Clinical Medical College, Yangzhou University, Yangzhou, China; ^2^ Institute of Epigenetics and Epigenomics, College of Animal Science and Technology, Yangzhou University, Yangzhou, China

**Keywords:** sperm DNA fragmentation, *in vitro* fertilization, intracytoplasmic sperm injection, fresh embryo transfer, frozen embryo transfer

## Abstract

**Purpose:**

To evaluate the effect of elevated sperm DNA fragmentation index (DFI) on fresh and frozen embryo transfer cycles.

**Methods:**

A retrospective study was performed with 549 fresh embryo transfer cycles and 1340 frozen embryo transfer cycles after *in vitro* fertilization/intracytoplasmic sperm injection (IVF/ICSI) from 2016 to 2021.

**Results:**

The statistical results of 549 fresh embryo transfer cycles showed that the delivery rate in the normal sperm DFI group (43.9% vs. 27.1%, *P* = 0.014) was significantly higher than that in the abnormal sperm DFI group, and there were no significant differences in the biochemical pregnancy rate (59.0% vs. 50.8%, *P* = 0.232), clinical pregnancy rate (53.1% vs. 40.7%, *P* = 0.072), or miscarriage rate (17.3% vs. 33.3%, *P* = 0.098) between the two groups. The results of 1340 frozen embryo transfer cycles showed that the biochemical pregnancy rate (57.9% vs. 45.6%, *P* = 0.006) and clinical pregnancy rate (50.3% vs. 40.7%, *P* = 0.027) in the normal sperm DFI group were significantly higher than those in the abnormal sperm DFI group. The delivery rate (40.9% vs. 33.3%, *P* = 0.074) and miscarriage rate (18.6% vs. 18.0%, *P* = 0.919) were not significantly different between the two groups.

**Conclusion:**

The increase of sperm DFI significantly reduced the delivery rate of fresh embryo transfer cycles and the biochemical pregnancy rate and clinical pregnancy rate of frozen embryo transfer cycles.

## Introduction

With the development of assisted reproductive technology (ART), new assisted reproductive methods and technologies continue to emerge. In addition to intrauterine insemination (IUI), the commonly used forms of ART include conventional *in vitro* fertilization (IVF) and intracytoplasmic sperm injection (ICSI) (1). In the assessment of male infertility, traditional semen analysis still has some limitations. Conventional semen analysis cannot detect subcellular sperm dysfunction, and sperm defects may lead to multiple IUIs and the inability to achieve pregnancy. Studies have shown no significant differences in routine semen parameters between fertile and infertile men, and even semen parameters in the “normal” range may contribute to differences in pregnancy outcomes (2, 3).

During spermatogenesis, germ cells undergo mitosis and meiosis to produce haploid sperm. Sperm are highly differentiated male germ cells consisting of a head, midsection and tail. The head contains the haploid genome, which is required for successful fertilization and is transferred to the oocyte. During sperm maturation, chromatin is highly condensed, the protamine process of sperm DNA is dysregulated or some germ cells fail to undergo apoptosis, thereby escaping the programmed phagocytosis process and forming defective mature sperm, often with an increased sperm DFI (4). Lin found that an increased DFI in hyperspermia was associated with increased miscarriage rates, but the effect was not significant (5). Zini (6) and Kenned (7) reported that sperm DNA damage resulted in a significant increase in the rate of miscarriage, while Virro (8) and Dar (9) found that the sperm DFI had no significant effect on the fertilization rate. Boe-Hansen found no significant effect of sperm DFI on biochemical pregnancy, clinical pregnancy, or embryo implantation (10). Oleszczuk reported that the sperm DFI significantly affects the live birth rate, high-quality embryo rate, and miscarriage rate (11). Green reported that the sperm DFI had no significant effect on the fertilization rate, blastocyst formation rate, implantation rate, ongoing pregnancy rate, or miscarriage rate (12).

The impact of sperm DNA fragmentation on IVF-embryo transfer (ET) is still controversial. In this study, semen specimens from men in IVF-ET cycles were collected, and the sperm DFI was detected to explore the effect of sperm DNA fragmentation on the clinical outcomes of IVF-ET.

## Materials and methods

### Study design and population

From January 2016 to April 2021, controlled ovarian hyperstimulation (COH) was performed at the Reproductive Medical Center of Subei People’s Hospital, and routine IVF/ICSI-ET was performed after egg retrieval. The exclusion criteria were as follows: (1) the woman’s B-ultrasound showed severe unilateral or bilateral hydrosalpinx; (2) the woman had polycystic ovary syndrome, high blood pressure, prolactinaemia, abnormal thyroid function, diabetes and other diseases; (3) the presence of uterine fibroids (diameter ≥ 4 cm or submucosal uterine fibroids), uterine malformation, endometriosis grades I-IV, intrauterine adhesions or other gynaecological complications; or (4) there were chromosomal abnormalities in the male/female. Regarding grouping, according to the analysis results obtained with the DFIViewer software, the men were divided into a normal sperm DFI group (DFI < 30%) and an abnormal sperm group (DFI ≥ 30%) according to their sperm DFI levels.

### Semen collection and routine analysis

The men abstained from sex for 2-7 days, and sperm were collected by masturbation. Routine semen processing analysis was performed according to the Laboratory Manual for Human Semen Examination and Processing, 5th Edition (13). The semen quality was analysed and recorded by using a computer-aided semen analyser (Beijing Suijia Medical Instrument) and checked manually. Freshly liquefied semen smears were air-dried and stained with a modified Pasteur method to record sperm morphology.

### Analysis of sperm DNA fragmentation rate

Sperm DNA fragmentation assay (SDFA) was performed using the Sperm Chromatin Analysis (SCSA) kit (Zhejiang Cellpro Biotech Co., Ltd., Ningbo, China) strictly in accordance with the product instructions (14). The detailed analysis process was as follows. First, appropriate volume of semen were added into 0.1 mL of solution A (TNE buffer, sperm dilution) and mixed. Then, 0.2 mL of solution B (acid solution of 0.1% Triton X-100, 0.15 mol/L NaCl, and 0.08 mol/L HCl, pH 1.2) were added and mixed. After standing for 30 s, 0.6 mL of acridine orange (AO) staining solution (6 μg/ml AO, 37 mmol/L citric acid, 126 mmol/L Na_2_HPO4, 1 mmol/L Na_2_EDTA, 0.15 mol/L NaCl, pH 6.0) was added and mixed. After sperm were stained for 3 min, the sperm DFI was detected by a flow cytometer (FACS Calibur, BD Bioscience, San Jose, CA, USA). A minimum of 5,000 sperm were acquired, and the data were analysed by the software (DFIView 2010 Alpha11.15, CellPro Biotech, Ningbo, China).The sperm DFI was expressed as the percentage of sperm with fragmented DNA compared to the total number of sperm. The variability of the replicate DFI measures was less than 5%.

### Semen optimization for IVF/ICSI

The semen was collected 2 hours before IVF, the men abstained for 2-3 days, and the semen was collected into a sterilized disposable wide-mouth collector. After checking the man’s name by fingerprint identification, the sperm spots were collected and placed in a 37°C incubator for incubation and liquefaction. After liquefaction, the semen was evaluated and recorded. The density gradient centrifugation method was used to optimize the semen. The specific operation steps were as follows. (1) The gradient centrifugation medium of 80% and 40% SpermGrad (Swedish Vitrolife Company) with two different concentrations was preheated in a 37°C incubator. (2) First, 1 ml of 80% high-concentration gradient centrifuge medium was added to the sterile conical centrifuge tube with a pipette, and then 1 ml of 40% low-concentration gradient medium was slowly added on top of it while being careful not to damage the interface between the two layers of gradient solution. Then 2 ml of liquefied semen was added (adding too much semen will cause overload and affect the separation effect). According to the specific conditions of the semen, the amount of gradient centrifugation fluid was adjusted, or the number of centrifuge tubes was increased. (3) The samples were placed in a centrifuge at 300-400 × g for 15 minutes, the supernatant and gradient solution were removed, and only approximately 0.5 ml of sperm pellet was taken from the bottom. Then, 3 ml of upstream insemination solution was added, mixed well, and transferred to a Falcon 1006 centrifuge tube. (4) The tube was placed into the centrifuge at 300-400 × g and centrifuged for 5 minutes. The supernatant was removed, the sperm precipitate that was visible at the bottom of the tube was obtained, and 0.5 mL of upstream insemination solution (IVF solution) was added. (5) The Falcon 1006 centrifuge tube was tilted at an angle of 30-45 degrees and placed in a 37°C, 6% CO_2_-saturated humidity incubator for upstream treatment. After 40 minutes, the upper sperm suspension was aspirated into another clean Falcon 1006 centrifuge tube to evaluate IVF use.

### IVF-ET

Drug-based ovarian stimulation was performed to stimulate follicle development until two or more follicles reached an average diameter of 18 mm. Then, a trigger was applied to induce final maturation of the developing oocytes, and approximately 36 hours after the trigger, oocytes were retrieved under transvaginal ultrasound guidance. On the day of egg retrieval, routine IVF was performed, and rescue ICSI (RICSI) was performed if no fertilization or low fertilization occurred. Progression through the cleavage stage was regularly monitored every day. After exclusion of transfer contraindications, ET or whole-embryo freezing was performed according to the condition of the woman and the embryos. Frozen embryo transfer was performed after frozen embryos were thawed. A blood test was performed 14-16 days after transplantation to measure the β-hCG level in peripheral blood to determine whether a biochemical pregnancy (more than 5.0 mIU/ml is diagnosed as biochemical pregnancy) was present. Luteal support was given in cases of biochemical pregnancy, and the presence of clinical pregnancy was determined 4-5 weeks after transplantation.

### Data statistics and analysis

Statistical analysis was performed using IBM SPSS Statistics 22.0 version 25 (IBM Corp., Armonk, NY, USA). Categorical variables were presented as frequencies and percentages, whereas continuous variables were reported as the means ± standard deviations (SDs) or as the medians and interquartile ranges (IQRs, 25th-75th percentile). The normality of the distribution of the variables was determined using the Kolmogorov-Smirnov test. Normally distributed data were expressed as the means and standard deviations, while the medians and IQRs were used for nonnormally distributed data. Correlation analysis was performed by the Pearson method or Spearman method The chi-square or Fisher’s exact test was used to compare categorical variables. *P* < 0.05 was considered statistically significant, and *P* < 0.01 was considered extremely significant.

## Results and analysis

### General statistics

A total of 1638 IVF/ICSI cycles were included in this study. The average age of the women was 30.56 years, the average age of the men was 31.79 years, and the average infertility period was 3.46 years. Among them, there were 1329 cases of conventional IVF cycles, 218 cases of ICSI cycles, 89 cases of IVF-RICSI cycles, and 2 cases of IVF-ICSI cycles. The Kolmogorov-Smirnov (K-S) test results were showed that the data did not obey a normal distribution (*P* < 0.01) (**Table 1**).

**Table 1:** Basic clinical data of the *in vitro* fertilization cycles.

	Median	Mean	Bias	Kurtosis	K-*S P* value
Female age (years)	30.00 (27.00-33.00)	30.56 ± 4.49	0.867	0.506	<0.001**
Male age (years)	31.00 (28.00-34.00)	31.79 ± 5.39	1.385	2.508	<0.001**
Infertility years	3.00 (2.00-4.50)	3.46 ± 2.62	2.236	7.816	<0.001**
Female BMI	22.30 (20.20-24.50)	22.56 ± 3.33	1.283	6.031	<0.001**
Male BMI	24.73 (22.46-27.36)	24.98 ± 3.82	0.482	3.263	0.001**
Abstinence days	4.00 (3.00-5.00)	4.16 ± 2.13	6.665	93.055	<0.001**
Semen volume (ml)	3.80 (2.70-5.10)	4.17 ± 2.01	0.894	0.839	<0.001**
Sperm concentration (10^6^/ml)	65.30 (35.51-102.0)	71.54 ± 47.65	1.374	6.205	<0.001**
Sperm motility (%)	58.00 (42.98-72.00)	56.14 ± 20.59	-0.466	-0.283	<0.001**
Sperm progressive motility (%)	48.00 (34.00-61.00)	46.86 ± 19.02	-0.317	-0.483	<0.001**
Sperm nonprogressive motility (%)	7.00 (4.00-14.00)	9.27 ± 7.10	1.378	4.664	<0.001**
Sperm immotility (%)	42.00 (28.00-57.11)	43.80 ± 20.37	0.455	-0.297	<0.001**
DFI (%)	14.79 (9.60-22.30)	17.50 ± 11.58	1.766	4.540	<0.001**
HDS (%)	6.05 (4.25-8.72)	7.24 ± 4.94	3.064	16.567	<0.001**

*(P < 0.01), **P < 0.01.

### Comparison of related parameters between the normal group and abnormal group in fresh embryo transfer cycles

A total of 549 fresh ET cycles in the IVF cycle were selected and grouped by the sperm DFI value, 490 cases were in the normal sperm DFI group (89.3%), and 59 cases were in the abnormal sperm DFI group (10.7%). The sperm concentration (*P* = 0.014), sperm motility (*P* < 0.001), and the percentage of sperm showing forward motility (*P* < 0.001) in the normal sperm DFI group were significantly higher than those in the abnormal sperm DFI group, and the abstinence days (*P* = 0.002) and sperm immobility percentage (*P* < 0.001) were significantly lower than those in the abnormal sperm DFI group. There were insignificant differences in female age (*P* = 0.222), male age (*P* = 0.208), infertility years (*P* = 0.941), female BMI (*P* = 0.917), male BMI (*P* = 0.204), endometrial thickness (*P* = 0.194), sperm high-staining HDS rate (*P* = 0.433), semen volume (*P* = 0.740), and sperm nonprogressive motility percentage (*P* = 0.056) between the two groups (**Table 2**).

**Table 2:** Comparison of related parameters between the normal DFI group and abnormal DFI group in fresh embryo transfer cycles.

	Normal group (DFI<30%)	Abnormal group (DFI≥30%)	Z value	*P* value
Abstinence days	4.00 (3.00-5.00)	4.50 (3.00-6.00)	-3.095	0.002**
Semen volume (ml)	3.50 (2.50-4.70)	3.35 (2.08-7.29)	-0.332	0.740
Sperm concentration (10^6^/ml)	66.70 (37.18-101.75)	51.25 (16.63-94.25)	-2.466	0.014*
Sperm motility (%)	57.00 (44.00-70.25)	33.50 (22.00-50.00)	-5.390	<0.001**
Sperm progressive motility (%)	50.00 (36.75-61.25)	27.00 (19.00-42.00)	-5.450	<0.001**
Sperm nonprogressive motility (%)	5.00 (3.00-8.00)	4.00 (2.00-8.00)	-1.913	0.056
Sperm immotility (%)	43.00 (30.00-57.00)	66.50 (50.00-78.00)	-5.372	<0.001**
HDS (%)	6.22 (4.37-8.86)	6.60 (4.19-10.15)	-0.784	0.433
Female age (years)	30.00 (28.00-33.00)	30.00 (28.00-35.00)	-1.221	0.222
Male age (years)	31.00 (28.00-34.00)	31.00 (28.00-35.00)	-1.260	0.208
Infertility years	3.00 (2.00-4.00)	3.00 (2.00-4.02)	-0.074	0.941
Female BMI	22.30 (20.10-24.50)	22.30 (20.88-25.00)	-0.104	0.917
Male BMI	25.02 (22.53-27.68)	24.22 (21.96-26.31)	-1.269	0.204
Intimal thickness (mm)	12.00 (10.00-14.00)	11.80 (9.60-14.00)	-1.299	0.194

*P < 0.05, **P < 0.01.

### Comparison of clinical outcomes between the normal and abnormal sperm DFI group in fresh embryo transfer cycles

Among the 549 cycles of fresh ET, there were 490 cases of normal sperm DFI, 289 cases of biochemical pregnancy (59.0%), 260 cases of clinical pregnancy (53.1%), 215 cases of childbirth (43.9%), and 45 cases of miscarriage (17.3%). There were 59 cases of abnormal sperm DFI, 30 cases of biochemical pregnancy (50.8%), 24 cases of clinical pregnancy (40.7%), 16 cases of childbirth (27.1%), and 8 cases of miscarriage (33.3%). The results showed that the biochemical pregnancy rate (59.0% vs. 50.8%, *P* = 0.232) and clinical pregnancy rate (53.1% vs. 40.7%, *P* = 0.072) of the normal sperm DFI group were higher than those of the abnormal sperm DFI group and that the abortion rate (17.3% vs. 33.3%, *P* = 0.098) was lower that of the normal sperm DFI group than in the abnormal sperm DFI group; however, the difference was not statistically significant. Additionally, the delivery rate (43.9% vs. 27.1%, *P* = 0.014) was significantly high in the normal sperm DFI group than in the abnormal sperm DFI group (**Table 3**).

**Table 3:** Comparison of pregnancy outcome rates between the normal and abnormal sperm DFI group in fresh embryo transfer cycles.

Pregnancy outcome	Normal group(DFI<30%)	Abnormal group (DFI≥30%)	χ2	*P* value
Biochemical pregnancy	59.0% (289/490)	50.8% (30/59)	1.431	0.232
Clinical pregnancy	53.1% (260/490)	40.7% (24/59)	3.234	0.072
Delivery	43.9% (215/490)	27.1% (16/59)	6.068	0.014*
Abortion	17.3% (45/260)	33.3% (8/24)	2.737	0.098

*P < 0.05, **P < 0.01.

### Comparison of related parameters between the normal group and abnormal group in frozen embryo transfer cycles

A total of 1340 frozen ET cycles were selected and grouped by the sperm DFI value. There were 1190 cases (88.8%) in the normal sperm DFI group and 150 cases (11.2%) in the abnormal sperm DFI group. There were statistically significant differences in female age (*P* = 0.041) and male age (*P* = 0.011) between the two groups, but the differences were not significant. Additionally, there was no significant difference in endometrial thickness (*P* = 0.786), number of transferred embryos (*P* = 0.353), sperm high-staining (HDS) rate (*P* = 0.76), or infertility years (*P* = 0.143) between the two groups (**Table 4**).

**Table 4:** Comparison of related parameters between the normal DFI group and abnormal DFI group in frozen embryo transfer cycles.

	Normal group (DFI<30%)	Abnormal group (DFI≥30%)	Z value	*P* value
Sperm DFI (%)	13.39 (9.25-18.7)	39.85 (32.32-46.82)	-19.983	0.000**
Intimal thickness (mm)	10.6 (9.0-12.0)	10.8 (9.45-12.3)	-0.272	0.786
Embryo transfer number	2.0 (1.0-2.0)	2.0 (1.0-2.0)	-0.929	0.353
HDS (%)	5.72 (4.10-8.58)	6.63 (4.15-10.40)	-1.772	0.76
Female age (years)	30.0 (28.0-34.0)	31.0 (28.0-34.0)	-2.041	0.041*
Male age (years)	31.0 (29.0-35.0)	32.0 (29.5-33.5)	-2.557	0.011*
Infertility years	3.08 (2.0-5.0)	4.25 (3.0-6.0)	-1.464	0.143

*P < 0.05, **P < 0.01.

### Impact of the sperm DFI on clinical outcomes in frozen embryo transfer cycles

Among 1340 frozen ET cycles, there were 1190 cases of normal sperm DFI, 689 cases (57.9%) of biochemical pregnancy, 598 cases (50.3%) of clinical pregnancy, 487 cases (40.9%) of delivery, and 111 cases (18.6%) of miscarriage. In the abnormal sperm DFI group, there were 69 cases (45.6%) of biochemical pregnancy, 61 cases (40.7%) of clinical pregnancy, 50 cases (33.3%) of delivery, and 11 cases (18.0%) of miscarriage. The statistical results showed that in the normal sperm DFI group, the biochemical pregnancy rate (57.9% vs. 45.6%, *P* = 0.006) and clinical pregnancy rate (50.3% vs. 40.7%, *P* = 0.027) were significantly higher than those of the abnormal sperm DFI group, and the delivery rate was higher than that of the abnormal sperm DFI group (40.9% vs. 33.3%, *P* = 0.074); however, the difference was not statistically significant, and the abortion rate of the normal sperm DFI group (18.6% vs. 18.0%, *P* = 0.919) was not significantly different from that of the abnormal DFI group (**Table 5**).

**Table 5:** Comparison of pregnancy outcome rates between normal and abnormal sperm DFI in frozen embryo transfer cycles.

Pregnancy outcome	Normal group (DFI<30%)	Abnormal group (DFI≥30%)	χ2	*P* value
Biochemical pregnancy	57.9% (689/1190)	46.0% (69/150)	7.667	0.006**
Clinical pregnancy	50.3% (598/1190)	40.7% (61/150)	4.897	0.027*
Delivery	40.9% (487/1190)	33.3% (50/150)	3.196	0.074
Abortion	18.6% (111/598)	18.0% (11/61)	0.010	0.919

*P < 0.05, **P < 0.01.

## Discussion

An increasing number of studies have found that the sperm DFI is closely related to semen parameters. In the current study, the analysis of 1638 IVF/ICSI cycles showed that the sperm DFI was significantly positively correlated with male age, indicating that the degree of sperm DNA fragmentation increased with age (**Supplementary Table 1**). This is consistent with the findings of Bellver (15), Ghanbarzadeh (16), Zhang (17), Gonzalez (18), Lu (19) and Belloc (20). The sperm DFI was significantly and positively correlated with abstinence days, semen volume, immotile sperm percentage, sperm high-staining HDS percentage and other parameters, suggesting that the increase in abstinence days may increase semen volume, but the immotile sperm percentage and sperm high-staining HDS percentage may increase at the same time. The expression of factors promoting sperm apoptosis leads to an increase in the sperm DFI value. The sperm DFI is significantly and negatively correlated with parameters such as sperm concentration, sperm motility, forward motility sperm percentage, and nonprogressive motility sperm percentage. Sperm concentration reflects the spermatogenic function of the testis to a certain extent. When spermatogenesis is in good condition, sperm DNA damage is reduced, and sperm motility, the percentage of motile sperm and the percentage of nonforward motile sperm all reflect the state of sperm to a certain extent. Motile sperm DNA integrity is good, and the sperm DNA fragmentation rate is low. Studies have found that the sperm DFI is positively correlated with obesity, and obesity-related abnormal lipid metabolism and reproductive function-altered hormones may lead to decreased sperm quality (21). This study found that the sperm DFI was negatively correlated with male BMI, which was inconsistent with the results of studies by Fariello (22), Tolouei (23), Ferigolo (24) and others showing that obesity led to an increased the sperm DFI. There was no significant correlation between sperm DFI and infertility years, suggesting that infertility years can be influenced by many factors. Studies with larger sample sizes may be needed to investigate the relationship between male BMI and the sperm DFI.

According to different sperm DFI values, groups were assembled to study the effect of the sperm DFI on the clinical outcome of IVF-fresh ET in ART. There were 549 fresh ET cycles including 490 cases (89.3%) in the normal sperm DFI group and 59 cases in the abnormal group (10.7%). The normal DFI group and abnormal group were similar in terms of female age, male age, years of infertility, female BMI, male BMI, endometrial thickness, semen volume, sperm high-staining HDS rate, and nonprogressive motile sperm percentage, among others. The lack of significant differences in these parameters suggests that there were no significant differences in the general data between the normal and abnormal groups in this study, reducing the influence of other factors on clinical outcomes. The sperm concentration, sperm motility, and percentage of forward motile sperm in the normal DFI group were significantly higher than those in the abnormal DFI group, and the percentage of immotile sperm and abstinence days in the normal DFI group were significantly lower than those in the abnormal DFI group. The results were consistent with Bieniek’s findings (25). Comparisons of the clinical outcomes between the normal and abnormal DFI groups showed that in the fresh ET cycle, the normal DFI group had a higher biochemical pregnancy rate (59.0% vs. 50.8%, *P* = 0.232) and clinical pregnancy rate (53.1% vs. 40.7%, *P* = 0.072) than the abnormal DFI group, but the difference was not significant; however, the delivery rate (43.9% vs. 27.1%, *P* = 0.014) was significantly higher in the normal DFI group than in the abnormal DFI group. Additionally, the abortion rate was lower than that in the abnormal group (17.3% vs. 33.3%, *P* = 0.098), but the difference was not significant.

Regarding the effect of the sperm DFI on the frozen ET cycles, a total of 1340 cases were included in this study. There was no statistically significant difference between the normal sperm DFI group and the abnormal sperm DFI group in terms of years of infertility, endometrial thickness, or the number of transferred embryos. There were statistically significant differences with the abnormal DFI group in terms of female age (30 vs. 31, *P* = 0.041) and male age (31 vs. 32, *P* = 0.011), but the difference was not large. Regarding the clinical outcomes, the biochemical pregnancy rate (57.9% vs. 46.0%, *P* = 0.006) and clinical pregnancy rate (50.3% vs. 40.7%, *P* = 0.027) of the normal DFI group were significantly higher than those of the abnormal group. The delivery rate (40.9% vs. 33.3%, *P* = 0.074) was higher in the abnormal DFI group, but the difference was not significant. Additionally, the abortion rate (18.6% vs. 18.0%, *P* = 0.919) of the normal DFI group was not significantly different from that of the abnormal group.

The results of this study showed that an abnormal sperm DFI in fresh embryo transfer cycles led to a significant decrease in the delivery rate, suggesting that a high sperm DFI will lead to abnormal embryo development, resulting in embryo or foetal loss before delivery. The differences in frozen embryo transfer cycle delivery rates were not significant, which may suggest that the effect of freezing damage on clinical outcomes should also be taken into account (e.g., the effect of freezing damage may be greater than the effect of sperm DFI on embryos at the beginning of fertilization). In the cryo-resuscitation cycle analysis, the biochemical pregnancy rate and clinical pregnancy rate were significantly different between the two groups, suggesting that the effect of the sperm DFI is significant between the embryo implantation and clinical pregnancy stages; however, the effect is not significant in delivery stages, indicating that once the embryo reaches a status of clinical pregnancy, the effect of sperm DFI is no longer significant. This information is helpful for guiding clinical practice. Studies have shown that the sperm DFI of men from couples experiencing habitual abortion is significantly higher than that of men without habitual abortion or men who are fertile (26–28). The effect of the sperm DFI on pregnancy outcomes in IVF-fresh ET suggests that paternal genes may begin to play a major role in later embryonic stages. The impact of the sperm DFI on IVF clinical outcomes is inconsistent. Selvam found that sperm DNA integrity may affect the outcome of conventional IVF-assisted pregnancy by affecting embryo quality (29). Zhang reported that the sperm DFI can be used as an indicator for evaluating pregnancy outcomes of ART-assisted pregnancy, and is one of the outcome predictors (30), but other results in the literature do not reveal a significant effect, making the sperm DFI not instructive for clinical practice (31). The results of this study suggest that although the semen on the egg retrieval day in the IVF-ET process is optimized by gradient centrifugation and/or upstream methods, the sperm is optimized, but the excessive DNA damage of the sperm will not only lead to a decrease in sperm quality but also have a significant effect on the delivery rate of fresh ET cycles and the biochemical and clinical pregnancy rates of frozen ET cycles.

## Data availability statement

The original contributions presented in the study are included in the article/**Supplementary Material**. Further inquiries can be directed to the corresponding authors.

## Ethics statement

The studies involving human participants were reviewed and approved by The studies involving human participants were reviewed and approved by Northern Jiangsu People’s Hospital ethics committee(2021ky068). The patients/participants provided their written informed consent to participate in this study, and the human tissues were obtained with informed consent. The patients/participants provided their written informed consent to participate in this study.

## Author contributions

HC and FL conceived the idea. CZ, FC and SZ collected the data and wrote the manuscript. HS, YJ, XW, CY and YS edited and revised the manuscript. ND,TX and KL checked the data. All authors listed have made a substantial, direct and intellectual contribution to the work, and approved it for publication.

## Funding

This study was funded by the Jiangsu postgraduate training innovation project (No. KYCX17_1888) and the National Natural Science Foundation of China (No. 81773013).

## Conflict of interest

The authors declare that the research was conducted in the absence of any commercial or financial relationships that could be construed as a potential conflict of interest.

## Publisher’s note

All claims expressed in this article are solely those of the authors and do not necessarily represent those of their affiliated organizations, or those of the publisher, the editors and the reviewers. Any product that may be evaluated in this article, or claim that may be made by its manufacturer, is not guaranteed or endorsed by the publisher.
