# Supplementary material for: Influence of sperm DNA fragmentation on the clinical outcome of in vitro fertilization-embryo transfer (IVF-ET)
Source: Front Endocrinol (Lausanne). 2022 Jul 14;13:945242. doi: 10.3389/fendo.2022.945242 (PMC9329669; doi:10.3389/fendo.2022.945242)
Supplement: Supplementary file 2 [file Table_1.docx]

| Supplementary Table 1 Correlation analysis between the sperm DFI and other parameters（*n*=1638） | | |
| --- | --- | --- |
|  | ρ值 | *P*值 |
| Male age (years) | 0.118 | ＜0.001** |
| Infertility years | 0.034 | 0.172 |
| Male BMI | -0.059 | 0.019* |
| Abstinence days | 0.097 | ＜0.001** |
| Semen volume (ml) | 0.067 | 0.007** |
| Sperm concentration (10^6^/ml) | -0.286 | ＜0.001** |
| Sperm motility (%) | -0.409 | ＜0.001** |
| Sperm progressive motility (%) | -0.429 | ＜0.001** |
| Sperm nonprogressive motility (%) | -0.052 | 0.037* |
| Sperm immotility (%) | 0.404 | ＜0.001** |
| HDS (%) | 0.172 | ＜0.001** |
| Note:**P*<0.05,***P*<0.01 | | |
